# A promising prognostic model for predicting survival of patients with HIV‐related diffuse large B‐cell lymphoma in the cART era

**DOI:** 10.1002/cam4.5957

**Published:** 2023-04-20

**Authors:** Juanjuan Chen, Yihua Wu, Zixin Kang, Shanfang Qin, Guangjing Ruan, Han Zhao, Xin Tao, Zhiman Xie, Jie Peng

**Affiliations:** ^1^ Department of Infectious Diseases Nanfang Hospital, Southern Medical University Guangzhou China; ^2^ Guangxi AIDS Diagnosis and Treatment Quality Control Center Longtan Hospital of Guangxi Zhuang Autonomous Region Liuzhou China; ^3^ Guangxi AIDS Clinical Treatment Center The Fourth People's Hospital of Nanning Nanning China; ^4^ Infectious Diseases Center Guangzhou Eighth People's Hospital, Guangzhou Medical University Guangzhou China

**Keywords:** diffuse large B‐cell lymphoma, human immunodeficiency virus, overall survival, prognostic model, red blood cell distribution width ratio

## Abstract

**Background:**

Optimization of risk stratification is important for facilitating prognoses and therapeutic decisions regarding diffuse large B‐cell lymphoma (DLBCL). However, a simple and applicable prognostic tool is lacking for individuals with human immunodeficiency virus (HIV)‐related DLBCL in the era of combined antiretroviral therapy (cART).

**Methods:**

This retrospective multicenter observational study included 147 HIV‐related DLBCL patients with histologically confirmed DLBCL from 2013 to 2020. The total group was divided into training (*n* = 78) and validation (*n* = 69) cohorts to derive the best prognostic score. Clinicopathological and characteristic biomarkers correlated with clinical outcomes were analyzed.

**Results:**

Age, Ann Arbor stage, lactate dehydrogenase (LDH) ratio, bulky disease, and red blood cell distribution width (RDW) ratio retained robust independent correlations with overall survival (OS) in multivariate analysis. A new and practical prognostic model was generated and externally validated, classifying patients into three categories with significantly different survival rates. Moreover, the new index outperformed the International Prognostic Index (IPI) score (area under the curve values of 0.94 vs. 0.81 in the training cohort and 0.85 vs. 0.74 in the validation cohort, C‐indices of 0.80 vs. 0.70 in the training cohort and 0.74 vs. 0.70 in the validation cohort, and integrated discrimination improvement values of 0.203 in the training cohort and 0.175 in the validation cohort) and was better at defining intermediate‐ and high‐risk groups. The calibration curves performed satisfactorily for predicting 3‐year OS in the training and validation cohorts.

**Conclusions:**

We developed and validated a simple and feasible prognostic model for patients with HIV‐related DLBCL that had more discriminative and predictive accuracy than the IPI score for risk stratification and individualized treatment in the cART era.

## Graphical abstract

Prognostic tool is unmet for human immunodeficiency virus (HIV)‐related DLBCL individuals in the era of combined antiretroviral therapy. In this retrospective multicenter observational study, we innovatively developed and externally validated a simple, feasible, and powerful new prognostic model in the HIV‐related DLBCL setting, which integrated host immunity. The novel index was superior to the traditional IPI score in terms of discrimination, risk stratification, and predictive accuracy, to inform individualized treatment decisions.

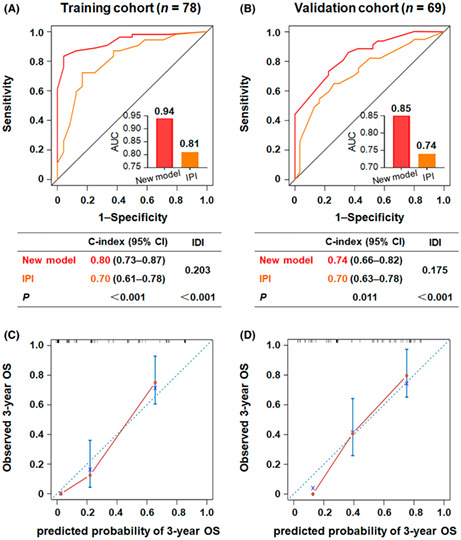

## 1 INTRODUCTION

Lymphomas have become a major cause of morbidity and mortality among individuals infected with human immunodeficiency virus (HIV) since the introduction of effective combined antiretroviral therapy (cART).^1^, ^2^, ^3^ Diffuse large B‐cell lymphoma (DLBCL), a leading HIV‐associated lymphoma, represents a phenotypically and genetically heterogeneous hematological malignancy.^4^ Compared with general patients, DLBCL patients are typically characterized by younger age, more advanced disease stage, more extranodal disease sites, and more aggressive presentation.^1^ In HIV‐negative populations, the prognosis has improved drastically over the past two decades, benefitting from the standard first‐line immune‐chemotherapeutic treatment and the development of new therapeutic approaches^5^; however, the outcome in patients with HIV‐related DLBCL is poor, especially in resource‐limited settings. First, the involved pathogenetic mechanisms of HIV‐associated lymphomas are heterogeneous, including HIV‐induced immunosuppression, genetic abnormalities, cytokine dysregulation, and immunologic derangement by Epstein–Barr virus and Kaposi sarcoma‐associated herpesvirus coinfection.^6^ Second, clinical trials of novel malignancy therapeutics have historically excluded patients with HIV.^7^ Third, the ability of available clinical prognostic indices to predict high‐risk groups is insufficient. Therefore, it is necessary to develop and validate new biomarkers for precise classification of HIV‐related DLBCL.

The original International Prognostic Index (IPI) score developed in 1993 is used as the benchmark for assessing the prognosis of HIV‐negative and HIV‐positive DLBCL patients,^8^, ^9^ but it is suboptimal in the identification of high‐risk subgroups, representing an unmet clinical need.^10^ Increasing research has focused on accurate risk stratification to develop individualized treatment strategies, such as the age‐adjusted IPI (aa‐IPI), National Comprehensive Cancer Network IPI (NCCN‐IPI), revised IPI (R‐IPI), international metabolic prognostic index, Elderly Prognostic Index (EPI), and relapsed/refractory IPI (RR‐IPI).^10^, ^11^, ^12^, ^13^ Nevertheless, prognostic stratification for HIV‐associated lymphomas relies heavily on general patients, limiting their generalizability to contemporary HIV cohorts.^14^, ^15^, ^16^ Emerging prognosis scores highlight the molecular features of the tumor microenvironment; however, they are unavailable and complicated in low‐resource areas. Thus, a simple, clinically applicable prognostic tool is needed to identify patients for consideration of novel prognostication and therapies in the HIV‐related DLBCL setting.

In this retrospective multi‐institutional study, we aimed to introduce clinical predictors of HIV‐ and lymphoma‐specific parameters and then establish and validate a prognostic model in the HIV‐related DLBCL setting, with an emphasis on clinical applicability and generalizability.

## 2 METHODS

### 2.1 Study population

**FIGURE 1:**
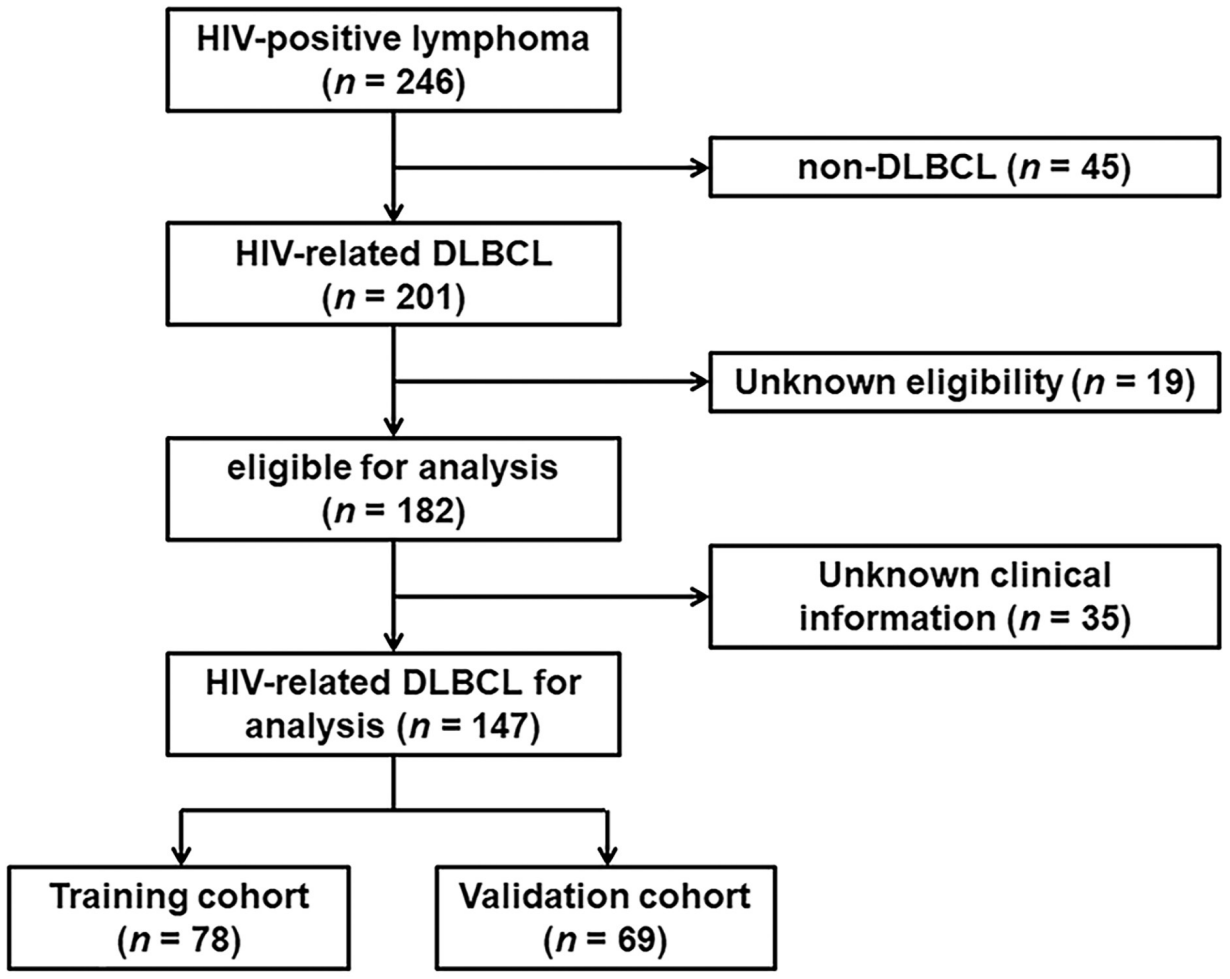
Flow diagram of patients' distributions.

This was a retrospective multicenter observational study. De novo HIV‐related DLBCL patients included in this study were diagnosed in the period of 2013–2020. The process of patient selection is illustrated in Figure 1. The pathological diagnosis of DLBCL was performed according to the 2008 World Health Organization (WHO) classification,^17^ established upon the initial lymph node or extranodal biopsy. The study was conducted among four institutions (Nanfang Hospital, Guangzhou, China; the Fourth People's Hospital of Nanning, Nanning, China; Guangzhou Eighth People's Hospital, Guangzhou, China; and Longtan Hospital of Guangxi Zhuang Autonomous Region, Liuzhou, China). Patients from Nanfang Hospital and the Fourth People's Hospital of Nanning were used as the training cohort, while patients from the other two hospitals were included for external validation.

### 2.2 Data collection

The baseline demographic and clinical characteristics and laboratory parameters were collected, including age at diagnosis, sex, lactate dehydrogenase (LDH), Eastern Cooperative Oncology Group performance status (ECOG PS), number of extranodal sites, Ann Arbor stage, IPI score, NCCN‐IPI score, B symptoms (night sweats, weight loss >10% over 6 months, and recurrent fever >38.3°C), bulky mass (>7.5 cm), complete blood cell count (CBC) variables [hemoglobin, platelet count (PLT), absolute lymphocyte count (ALC), absolute monocyte count (AMC), lymphocyte to monocyte ratio (LMR), and red blood cell distribution width (RDW) ratio], prior history of HIV, CD4 count, CD4/CD8 ratio, cART condition, radiological examinations, immunochemotherapy, cause of death, survival months, cell of origin, CD20 expression, cytogenetic abnormalities, Ki‐67 expression, Epstein–Barr virus‐encoded small RNA (EBER) status, and treatment outcomes.

### 2.3 Clinical assessments

The primary endpoint of our study was overall survival (OS), defined as the time from diagnosis of HIV‐related DLBCL to the last follow‐up or death from any cause. Computed tomography (CT) or 18F‐fluorodeoxyglucose positron emission tomography/computed tomography (FDG PET/CT) was performed for radiological evaluation. Brain magnetic resonance imaging (MRI) was used to assess central nervous system involvement.

### 2.4 Construction of the new prognostic model

To develop a new prognostic score, the patients were split into training and validation cohorts. Risk factors were based on previous studies and were routinely available in clinical practice. The new model was derived through univariate and multivariate analyses of the training set. Comparisons between scores were investigated by the area under the curve (AUC) of the receiver operating characteristic (ROC) curve, Harrell's concordance index (C‐index), and integrated discrimination improvement (IDI).

### 2.5 Statistical analysis

Survival curves were calculated using Kaplan–Meier estimators and compared using the log‐rank test. The optimal cutoff values were calculated from the ROC curve. Cox regression models were used to evaluate the relationship between demographic and clinical variables and OS. Hazard ratios (HRs) and 95% confidence intervals (CIs) were calculated to assess the strength of the relationships. For the multivariate Cox regression analysis, significant variables (*p* < 0.05) in the univariate analyses were considered. The prognostic index was established with coefficients for the independent prognostic factors weighted by the multivariate analysis. AUCs according to Cox regression, C‐index, and IDI were calculated through R (version 4.1.2. https://www.r‐project.org/) to determine the best model fit and to assess model performance. Statistical analyses were performed using SPSS version 26.0 software. A two‐sided *p* value <0.05 was considered statistically significant.

## 3 RESULTS

### 3.1 Patient features and survival

**FIGURE 2:**
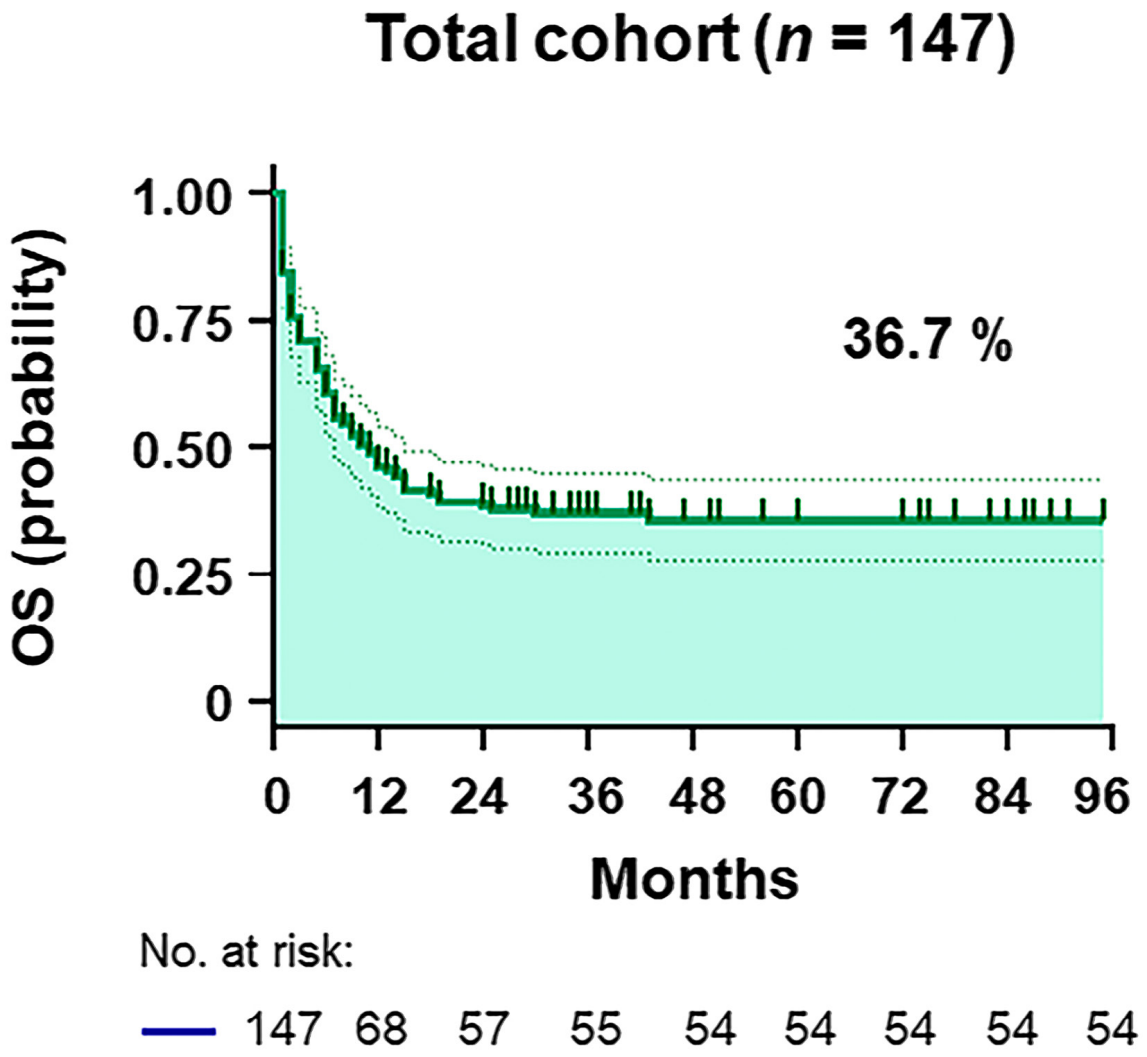
Survival analysis. Kaplan–Meier estimate of overall survival in the total cohort.

The clinical characteristics of the patients from the four hospitals were merged into a dataset comprising 147 HIV‐related DLBCL patients (Figure 1). The training and validation cohorts consisted of 78 and 69 patients, respectively, with similar baseline features, as shown in Table 1 and Table S1. With a median follow‐up duration of 22.4 (interquartile range [IQR], 1–95) months, the OS was 36.7% in all patients (Figure 2 and Figure S1). The median age was 49 (IQR, 18–74) years old, with 83.0% being male and 25.2% having a prior history of HIV infection (Table 1). As shown in Table 1, 71 (48.3%) patients with HIV‐related DLBCL had an IPI of 3–5 at diagnosis, 33 (22.4%) were aged >60 years old, 109 (74.2%) presented an LDH ratio > 1, 83 (56.5%) had an ECOG PS > 1, 81 (55.1%) were Ann Arbor stage III/IV, and 39 (26.5%) had extranodal disease >1 site in the whole cohort. The optimal cutoff points of the RDW ratio and CD4 T‐cell count were determined by ROC analysis, with values of 0.9 and 180 cells/μL, respectively (Figure S2). An RDW ratio ≥ 0.9 was documented in 96 (65.3%) patients, and the mean CD4 T‐cell count was 200 (IQR, 3–1089) cells/μL (Table 1). Overall, there were no substantive differences between the training and validation cohorts.

**TABLE 1:** Baseline characteristics of patients with HIV‐related DLBCL.

Variables	Total (*n* = 147)	Training (*n* = 78)	Validation (*n* = 69)	*p*
Sex				0.907
Male	122 (83.0)	65 (83.3)	57 (82.6)	
Age (years)				0.811
≤40	41 (27.9)	20 (25.6)	21 (30.4)	
41–60	73 (49.7)	40 (51.3)	33 (47.8)	
61–75	33 (22.4)	18 (23.1)	15 (21.7)	
≥75	0 (0.0)	0 (0.0)	0 (0.0)	
Age, median (range)	49 (18–74)	49 (24–74)	48 (18–74)	0.309
LDH ratio				0.998
≤1	38 (25.9)	20 (25.6)	18 (26.1)	
1–3	62 (42.2)	33 (42.3)	29 (42.0)	
>3	47 (32.0)	25 (32.1)	22 (31.9)	
ECOG PS				0.324
>1	83 (56.5)	47 (60.3)	36 (52.2)	
Extranodal sites				**0.033**
>1	39 (26.5)	15 (19.2)	24 (34.8)	
Stage				0.511
III–IV	81 (55.1)	41 (52.6)	40 (58.0)	
IPI				0.161
Low	45 (30.6)	23 (29.5)	22 (31.9)	
Low‐intermediate	31 (21.1)	19 (24.4)	12 (17.4)	
High‐intermediate	39 (26.5)	24 (30.8)	15 (21.7)	
High	32 (21.8)	12 (15.4)	20 (29.0)	
B Symptoms				0.346
Present	37 (25.2)	17 (21.8)	20 (29.0)	
Bulky mass				**0.002**
>7.5 cm	58 (39.5)	40 (51.3)	18 (26.1)	
RDW ratio				0.983
≥0.9	96 (65.3)	51 (65.4)	45 (65.2)	
Prior history of HIV				0.167
Yes	37 (25.2)	16 (20.5)	21 (30.4)	
CD4 count (cells/μL)				0.466
<180	75 (51.0)	42 (53.8)	33 (47.8)	

*Note*: *p* value was calculated by Pearson Chi‐Square test between patients in the training and validation cohorts.

Bold value indicates *p* < 0.05.

Abbreviations: ECOG PS, Eastern Cooperative Oncology Group performance status; IPI, International Prognostic Index; LDH, lactate dehydrogenase; RDW, red blood cell distribution width.

### 3.2 Independent prognostic factors for OS

**FIGURE 3:**
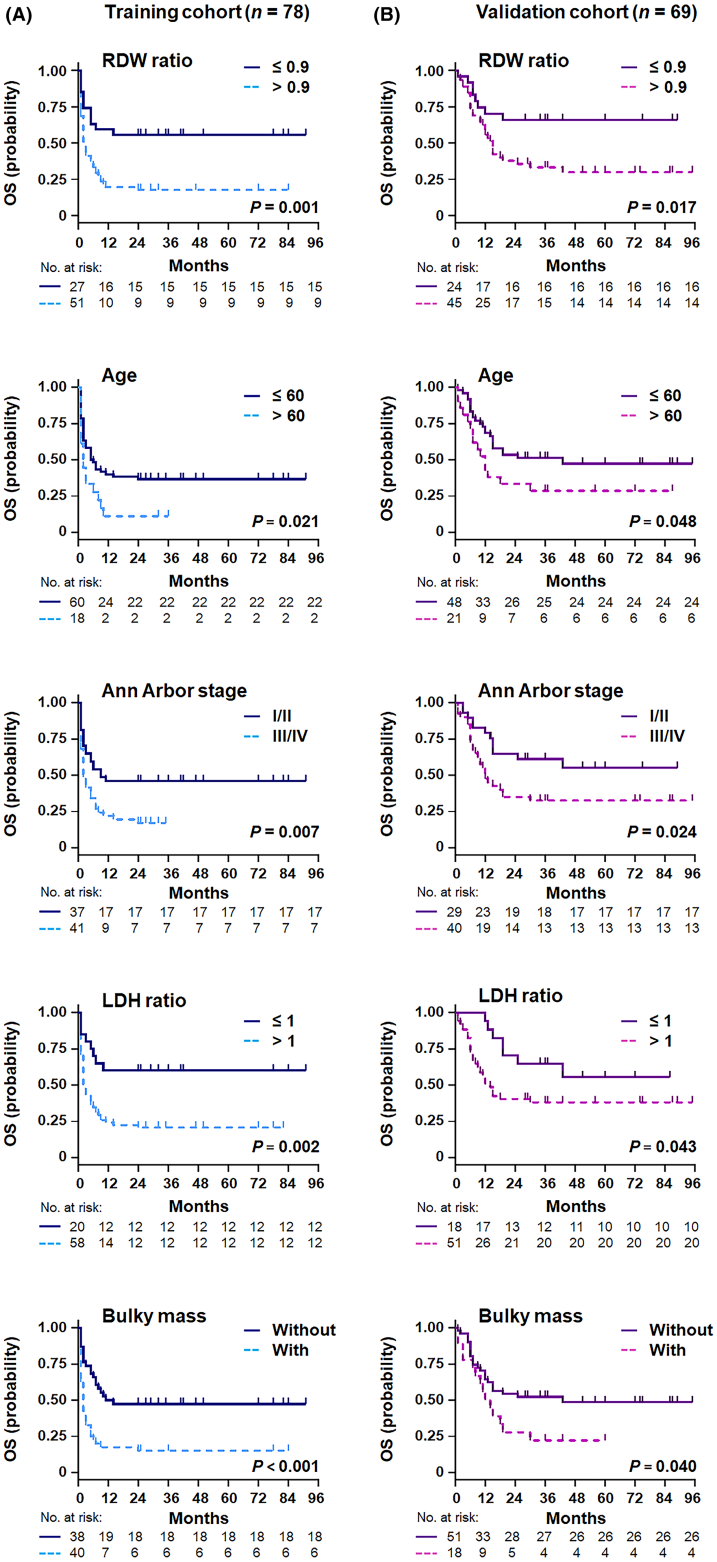
Overall survival according to the independent predictors in patients with HIV‐related DLBCL. Kaplan–Meier curves for RDW ratio, age, Ann Arbor stage, LDH ratio, and bulky mass in the training (A) and validation (B) cohorts.

By univariate analysis, the clinical factors associated with OS were age, LDH ratio, ECOG PS, Ann Arbor stage, number of extranodal sites, bulky mass, and RDW ratio in the training cohort (Table 2). All predictors correlated with survival in univariate analyses (*p* < 0.05) were kept for multivariate analysis, including the CD4 count (Table 2 and Figure S3A). Prognostic factors independently and significantly associated with survival outcomes in multivariate analysis included older age, higher LDH ratio, advanced Ann Arbor stage, bulky disease, and elevated RDW ratio (Table 2 and Figure 3A).

**TABLE 2:** Univariate and multivariate analyses of overall survival in the training and the validation cohorts of HIV‐related DLBCL individuals.

Variables	Univariate				Multivariate			
	Training		Validation		Training		Validation	
	HR (95% CI)	*p*	HR (95% CI)	*p*	HR (95% CI)	*p*	HR (95% CI)	*p*
Sex (male)	0.868 (0.437–1.726)	0.687	1.672 (0.653–4.281)	0.284				
Age (>60)	1.863 (1.033–3.359)	**0.039**	2.106 (1.101–4.026)	**0.024**	1.888 (1.024–3.482)	**0.042**	1.493 (0.736–3.029)	0.267
LDH ratio (>1)	2.849 (1.338–6.070)	**0.007**	2.203 (1.009–4.807)	**0.047**	2.498 (1.152–5.416)	**0.020**	1.590 (0.698–3.621)	0.269
ECOG PS (≥2)	2.464 (1.333–4.554)	**0.004**	1.755 (0.925–3.330)	0.085				
Extranodal sites (>1)	2.027 (1.077–3.815)	**0.028**	1.961 (1.034–3.717)	**0.039**				
Stage (III/IV)	2.004 (1.148–3.498)	**0.014**	2.128 (1.075–4.212)	**0.030**	1.980 (1.117–3.509)	**0.019**	1.557 (0.759–3.195)	0.228
B Symptoms (present)	1.135 (0.597–2.157)	0.700	0.911 (0.444–1.871)	0.800				
Bulky mass (>7.5 cm)	2.559 (1.456–4.498)	**0.001**	2.903 (1.531–5.506)	**0.001**	2.631 (1.477–4.688)	**0.001**	2.144 (1.046–4.397)	**0.037**
RDW ratio (>0.9)	2.594 (1.357–4.958)	**0.004**	2.465 (1.131–5.371)	**0.023**	2.183 (1.127–4.226)	**0.021**	1.862 (0.824–4.208)	0.135
Prior history of HIV (Yes)	0.868 (0.447–1.686)	0.677	1.230 (0.631–2.398)	0.542				
CD4 count (<180)	1.628 (0.934–2.839)	0.086	2.014 (1.061–3.822)	**0.032**	1.490 (0.721–3.102)			0.290
CD4/CD8 ratio (<1)	1.682 (0.232–12.168)	0.603	5.010 (0.687–36.523)	0.077				
PLT < 100	1.012 (0.365–2.808)	0.981	2.740 (1.135–6.617)	**0.020**				
ALC < 1000/μL	1.497 (0.876–2.559)	0.137	0.917 (0.471–1.786)	0.799				
AMC ≥ 630/μL	0.948 (0.508–1.770)	0.867	1.135 (0.582–2.212)	0.710				
LMR <2.25	1.066 (0.625–1.819)	0.815	1.312 (0.699–2.461)	0.397				
Anemia	1.704 (0.938–3.098)	0.077	1.479 (0.759–2.881)	0.247				

Abbreviations: ALC, absolute lymphocyte count; AMC, absolute monocyte count; ECOG PS, Eastern Cooperative Oncology Group performance status; LDH, lactate dehydrogenase; LMR, lymphocyte/monocyte ratio; PLT, platelet; RDW, red blood cell distribution width.

Bold value indicates *p* < 0.05.

### 3.3 Development of the new prognostic model

**FIGURE 4:**
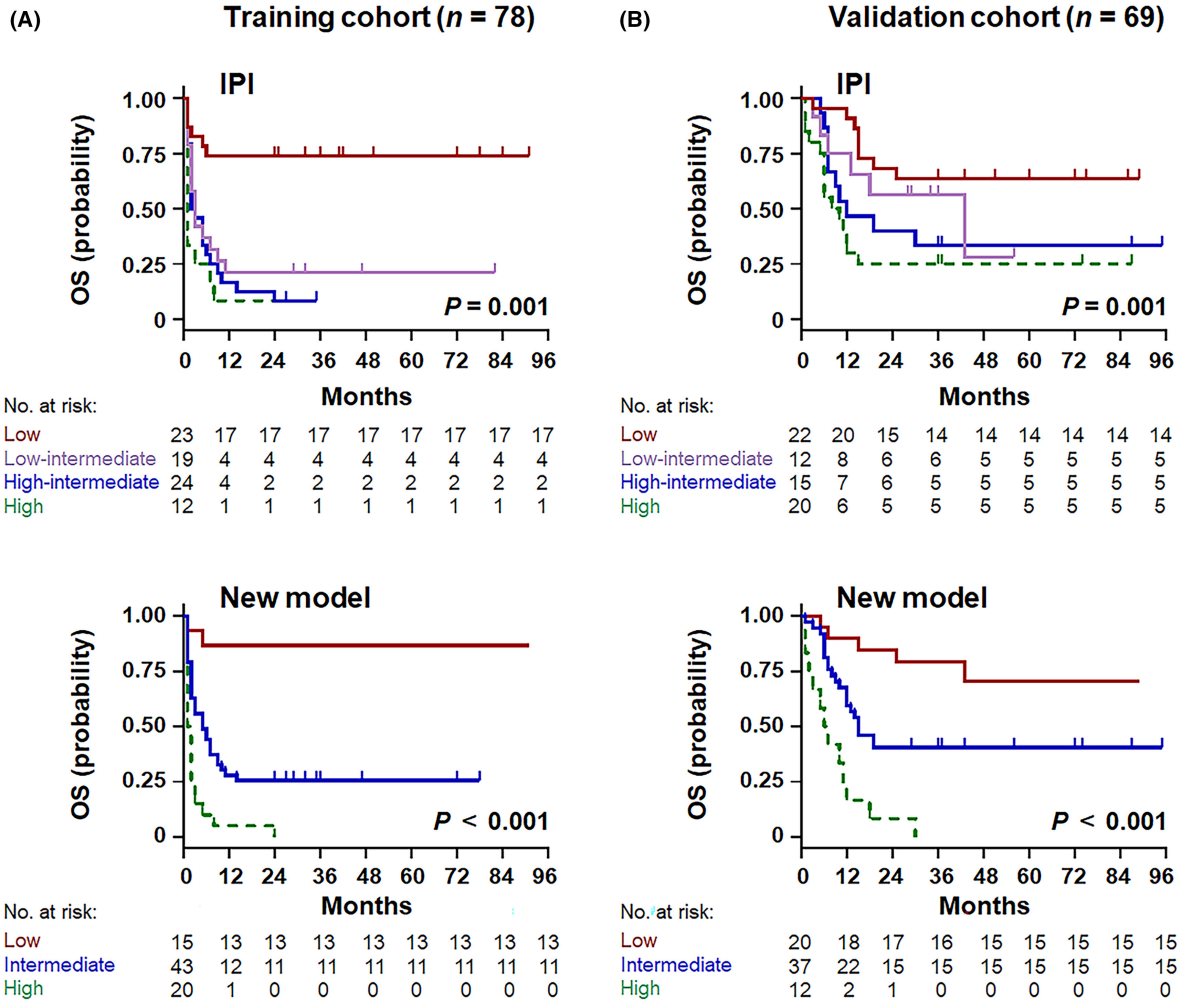
New prognostic model for risk stratification of overall survival in the HIV‐related DLBCL setting. Kaplan–Meier plots comparing the novel predictive model with the IPI score in the training (A) and validation (B) cohorts.

The five independent risk factors were used to construct a new prognostic index for HIV‐related DLBCL. Each variable was scored as 1, considering the hazard ratio.^18^ Noting excellent separation in the Kaplan–Meier curve (Figure 4A), three risk groups were identified in the training cohort, namely the low‐ (scores: 0–1; 19.2%), intermediate‐ (scores: 2–3; 55.1%), and high‐risk groups (scores: 4–5; 25.6%), with OS rates corresponding to 86.7% (95% CI 64.1–94.4%, *p* < 0.001), 25.6% (95% CI 13.4–32.7%, *p* < 0.001), and 0.0% (95% CI 0.9–5.4%, *p* < 0.001), respectively. The novel predictive model displayed impressive performance for survival outcomes, with accurate separation of the three groups and better discrimination for the intermediate‐ and high‐risk groups than the IPI score (Figure 4A).

### 3.4 Validation of the new prognostic model

In the external validation cohort, age, LDH ratio, Ann Arbor stage, number of extranodal sites, bulky mass, RDW ratio, and CD4 count were predictors of shorter OS (Table 2; Figure 3B; Figure S3B), demonstrating promising agreement with the training cohort. According to the new prognostic index, 29.0% of patients were classified as low risk, 53.6% as intermediate risk, and 17.4% as high risk, which corresponded to OS rates of 75.0% (95% CI 54.5–84.2%, *p* = 0.013), 40.5% (95% CI 31.0–58.0%, *p* < 0.001), and 0.0% (95% CI 4.1–13.6%, *p* < 0.001), respectively (Figure 4B). Compared with the conventional IPI score, the novel multivariate model represented superior intergroup (intermediate risk/high risk) differences for predicting OS.

### 3.5 Sensitivity analysis

**FIGURE 5:**
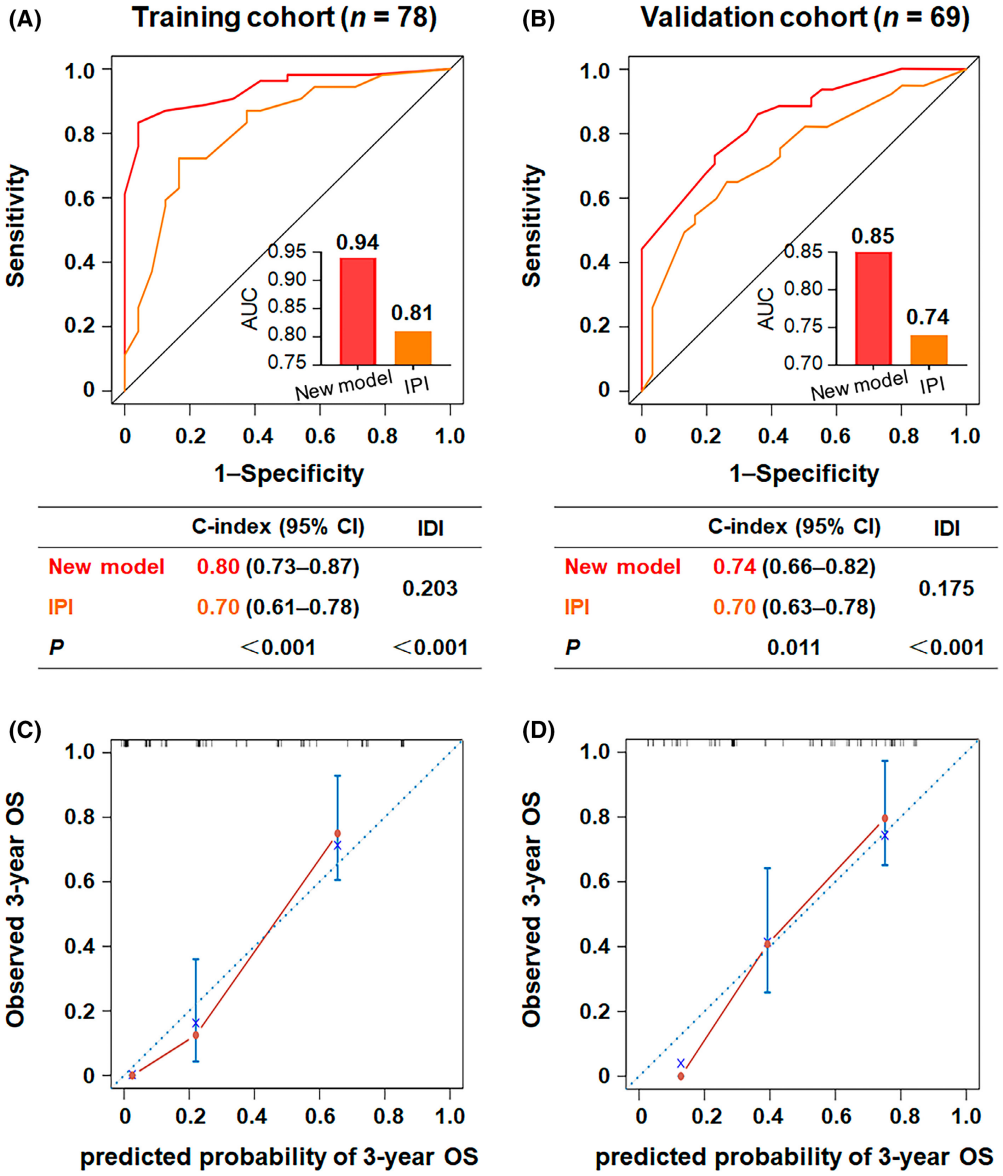
Comparison of the predictive performance. Receiver operating characteristic (ROC) curves, area under the curve (AUC), C‐indices, and integrated discrimination improvement (IDI) were used to assess the predictive performance of the new prognostic model compared with the IPI score in the training (A) and validation (B) cohorts. The calibration curves of the new index for predicting 3‐year overall survival in the training (C) and validation (D) cohorts.

The new risk assessment model consistently outperformed the IPI score for predicting OS in both the training and validation cohorts (Figure 5). ROC analysis (Figure 5A,B) showed that the novel index had higher predictive accuracy for all patients than the IPI (AUC in the training set: 0.94 vs. 0.81, *p* < 0.001; AUC in the validation set: 0.85 vs. 0.74, *p* < 0.001). Similarly, the Harrell's C‐indices of the new calculator were higher than those of the IPI model in the training (0.80 vs. 0.70, *p* < 0.001) and validation (0.74 vs. 0.70, *p* = 0.011) cohorts, respectively. Furthermore, the estimated IDI gained by the new prognostic model in the training and validation cohorts was 0.203 (*p* < 0.001) and 0.175 (*p* < 0.001), respectively, providing better predictive ability than the IPI. In addition, the calibration curves for the probability of 3‐year OS showed consistently favorable agreement between the actual observed outcome and the prediction by the new model in both the training (Figure 5C) and validation cohorts (Figure 5D).

## 4 DISCUSSION

In this study, we created a simple and powerful new prognostic model on the basis of age, Ann Arbor stage, LDH ratio, bulky disease, and RDW ratio. The novel multivariate risk index is superior to the traditional IPI score in terms of discrimination, risk stratification, and predictive accuracy in patients with HIV‐related DLBCL. In addition to the remarkable advantage and clinical utility, the prognostic calculator has the potential benefit of informing accurate treatment decisions.

The IPI is the most widely used score for non‐Hodgkin lymphoma but lacks the ability to identify a high‐risk population among patients with DLBCL.^18^ There is now a general consensus that HIV‐infected lymphoma patients have a higher risk of death than the general population^2^, ^19^; accordingly, optimization of risk assessment is important for facilitating prognoses and therapeutic decisions. We demonstrated that tumor load (high LDH ratio and bulky mass), invasive potential (advanced Ann Arbor stage), host features (increased age), and immune activation (elevated RDW ratio) were independently associated with inferior survival in HIV patients. Consistent with data from large population‐based studies,^11^, ^20^ age, stage, and LDH retain major prognostic impact, providing opportunities for identifying risk individuals accurately. Therefore, our new model involved these principal adverse factors of IPI with clinical rationale. In contrast, the clinical utility of ECOG PS and extranodal invasion, useful for the IPI score, was not optimal in our HIV‐related DLBCL cohort. Generally, higher propensities of extranodal disease and aggressive presentation could confound their predictive performance in patients with HIV‐related DLBCL.^1^, ^6^ Performance status might be limited by the small sample size and the subjectivity and fluctuation of registrar.^11^ Furthermore, the prognostic impact of extranodal involvement has been questioned, especially in the era of rituximab.^20^, ^21^ Future studies should distinguish primary from secondary extranodal involvement and determine the association of biological behavior with the risk of central nervous system invasion or recurrence.^22^, ^23^

Of note, the RDW ratio, as a novel independent poor survival predictor of HIV‐positive DLBCL, was integrated into the prognostic index first. As a complete blood cell count parameter and an emerging biomarker, the RDW ratio is involved in many neoplastic and nonneoplastic pathophysiological conditions, such as carcinogenesis, tumor progression, cardiovascular disease, pulmonary disease, and diabetes.^18^, ^24^, ^25^, ^26^, ^27^ Additionally, the RDW ratio, associated with inflammation, is being promoted as a prognostic variable in various neoplastic diseases, such as hematological malignancies and solid tumors.^28^ Similarly, this readily available clinical detail appears to be an attractive marker in HIV‐negative DLBCL patients.^18^ There is increasing evidence that the tumor microenvironment and host immunity play an important role in the context of DLBCL. The mechanism is not fully understood and might be correlated with systemic inflammation, impaired nutritional status, bone marrow malfunction, higher tumor burden, and impaired antineoplastic immunity.^29^, ^30^ To the best of our knowledge, this is the first study to explore the correlation between the RDW ratio and adverse survival in newly diagnosed HIV‐related DLBCL.

Prior studies have reported the adverse prognostic significance of bulky disease in DLBCL, with no significant association with IPI and age.^18^, ^31^, ^32^ However, the role of this parameter in the HIV setting is uncertain and merits study. In the current study, we validated the independent inverse correlation between tumor burden and clinical outcome. Recent works have shown that baseline metabolic tumor volume is a promising predictor of survival in DLBCL, regardless of the measurement method.^11^, ^33^ As bulky mass partly measured disease burden, the inclusion of bulky mass improved HIV‐related DLBCL risk stratification in our study. Unfortunately, limited by the retrospective design, this factor needs to be interpreted with caution in the rituximab era.^34^ Additional validation using clinical trial data could further explain the prognostic impact of bulky disease adequately.

Immunosuppression plays a notable role in the development of lymphomas,^35^ but the prognostic impact of CD4 in HIV‐associated lymphomas remains controversial.^36^ In this study, the CD4 count in multivariate analysis (Table 2) and different cART drug classes in the log‐rank test (Table S1) had no statistical significance, indicating that HIV infection was not a pivotal competing risk in the era of cART. A few groups have reported the prognostic significance of CD4 in HIV‐associated lymphomas.^14^ Conversely, multiple independent studies have demonstrated that HIV‐specific indicators, such as low CD4 count, prior history of HIV, and HIV viral load, are not correlated with inferior outcomes in the current treatment era.^37^, ^38^ The popularization of cART offers superior virological suppression and immune reconstitution, which is beneficial for HIV patients to receive the same intensive curative therapies as general patients.^39^, ^40^ Clinical cohort trials are further needed to uncover comparable prognostic information for CD4 T cells.

The new prognostic model integrates information about host factors and disease burden in a simple and practical index using five easily obtained clinical variables. The calculator is also credible and was developed and externally validated in the real‐world setting of HIV‐related DLBCL, with consistent calibration and discrimination. Moreover, the novel prognostic tool provides the feasible advantage of accurate risk assessment for the poor survival subgroup. Characterizing the personalized prognosis of HIV‐related DLBCL is important to better identify the highest‐risk patients, which may facilitate clinicians in incorporating prognostic stratification into new therapeutic approach decisions, although any treatment modifications need to be examined in prospective studies.^10^, ^41^

The limitations of this study include its retrospective design with inherent biases. In addition, the causes of survival differences at different centers in China, even between Chinese and international cohorts, are unclear. Furthermore, this real‐world study comprised low‐ and middle‐income populations with lower popularization of rituximab, partly owing to the scarcities of trained specialists, diagnostic facilities, treatment resources, supportive care, and patient affordability in developing countries. The same dilemma exists in other countries and regions with insufficient or imbalanced resources, potentially reflecting the disparities in treatment paradigms and socioeconomic conditions. Thus, additional evaluation and recalibration of the new calculator could be needed in future prospective clinical trials with uniform chemoimmunotherapy. Nevertheless, the novel multivariate risk index reflects the real‐life setting and is an objective tool for stratification in clinical practice, which might assist in decision‐making of subsidy strategies by the government.

In summary, we present an externally validated, easy‐to‐apply and more feasible novel prognostic index for the clinical practice of HIV‐related DLBCL populations in the real‐world setting. The new model was based on disease characteristics and provided precise prognostic stratification, which could be a promising tool for tailoring appropriate risk‐adapted therapies and surveillance strategies.

## AUTHOR CONTRIBUTIONS

**Juanjuan Chen:** Conceptualization (lead); data curation (lead); formal analysis (lead); funding acquisition (lead); validation (lead); visualization (lead); writing – original draft (lead); writing – review and editing (lead). **Yihua Wu:** Formal analysis (equal); investigation (lead); methodology (lead); software (equal); visualization (equal); writing – original draft (equal). **Zixin Kang:** Investigation (equal); methodology (equal); validation (equal). **Shanfang Qin:** Data curation (supporting); investigation (supporting). **Guangjing Ruan:** Data curation (supporting); investigation (supporting). **Han Zhao:** Validation (supporting). **Xin Tao:** Data curation (supporting). **Zhiman Xie:** Conceptualization (equal); data curation (equal); supervision (equal); writing – review and editing (supporting). **Jie Peng:** Conceptualization (equal); data curation (equal); funding acquisition (equal); project administration (equal); supervision (lead); writing – review and editing (equal).

## FUNDING INFORMATION

This project was supported by grants from the National Natural Science Foundation of China (81971949), Clinical Research Program of Nanfang Hospital, Southern Medical University (2018CR026), Natural Science Foundation of Guangdong Province, China (2023A1515030252, 2018A030313571, and 2017A030310649), and Major Science and Technology Special Project of Nanning (20193008).

## CONFLICT OF INTEREST STATEMENT

The authors declare that they have no competing interests.

## ETHICS STATEMENT

This study was conducted in accordance with the Declaration of Helsinki and was approved by the respective ethics committees. All patients provided their written informed consent including analysis of data.

## Supporting information


Data S1

## Data Availability

The datasets used and/or analyzed during the current study are available from the corresponding author on reasonable request.
