# Supplementary material for: A promising prognostic model for predicting survival of patients with HIV‐related diffuse large B‐cell lymphoma in the cART era
Source: Cancer Med. 2023 Apr 20;12(11):12470–81. doi: 10.1002/cam4.5957 (PMC10278482; doi:10.1002/cam4.5957)
Supplement: Supplementary file 1 — Data S1 [file CAM4-12-12470-s001.docx]

**A promising prognostic model for predicting survival of patients with HIV-related diffuse large B-cell lymphoma in the cART era**

Juanjuan Chen^1^ | Yihua Wu^1^ | Zixin Kang^1^ | Shanfang Qin^2^ | Guangjing Ruan^3^ | Han Zhao^1, 4^ | Xin Tao^1^ | Zhiman Xie^3^ | Jie Peng^1^

^1^Department of Infectious Diseases, Nanfang Hospital, Southern Medical University, Guangzhou, China

^2^Guangxi AIDS Diagnosis and Treatment Quality Control Center, Longtan Hospital of Guangxi Zhuang Autonomous Region, Liuzhou, China

^3^Guangxi AIDS Clinical Treatment Center, the Fourth People's Hospital of Nanning, Nanning, China

^4^Infectious Diseases Center, Guangzhou Eighth People's Hospital, Guangzhou Medical University, Guangzhou, China

Correspondence:

Jie Peng

pjie138@163.com

Zhiman Xie

20060830xzm@163.com

^1^Department of Infectious Diseases, Nanfang Hospital, Southern Medical University, Guangzhou 510515, China

^3^Guangxi AIDS Clinical Treatment Center, the Fourth People's Hospital of Nanning, Nanning 530023, China

Juanjuan Chen, Yihua Wu and Zixin Kang are co-first authors on this work. Jie Peng and Zhiman Xie are joint senior authors.

**SUPPLEMENTARY TABLE**

**SUPPLEMENTARY TABLE 1** The treatment and outcome information of HIV-related DLBCL setting

| Variables | Total  (*n* = 147) | Training  (*n* = 78) | Validation  (*n* = 69) | *P* |
| --- | --- | --- | --- | --- |
| **Cell of origin** |  |  |  | 0.066 |
| GCB | 80 (54.4) | 44 (56.4) | 36 (52.2) |  |
| Non-GCB | 24 (16.3) | 15 (19.2) | 9 (13.0) |  |
| Inconclusive | 43 (29.3) | 19 (24.4) | 24 (34.8) |  |
| **CD20** |  |  |  | 0.350 |
| Positive | 94 (63.9) | 48 (61.5) | 46 (66.7) |  |
| Negative | 1 (0.7) | 0 (0.0) | 1 (1.4) |  |
| Inconclusive | 52 (35.4) | 30 (38.5) | 22 (31.9) |  |
| **Cytogenetic abnormalities** |  |  |  | **0.020** |
| DE | 16 (10.9) | 6 (7.7) | 10 (14.5) |  |
| Non-DE | 41 (27.9) | 12 (15.4) | 29 (42.0) |  |
| Inconclusive | 90 (61.2) | 60 (76.9) | 30 (43.5) |  |
| **EBER status** |  |  |  | 0.708 |
| Positive | 18 (12.2) | 10 (12.8) | 8 (11.6) |  |
| Negative | 25 (17.0) | 15 (19.2) | 10 (14.5) |  |
| Inconclusive | 104 (70.7) | 53 (67.9) | 51 (73.9) |  |
| **Ki-67 expression** |  |  |  | 0.574 |
| > 80% | 67 (45.6) | 38 (48.7) | 29 (42.0) |  |
| < 80% | 19 (12.9) | 8 (10.3) | 11 (16.0) |  |
| Inconclusive | 61 (41.5) | 32 (41.0) | 29 (42.0) |  |
| **cART** |  |  |  | **0.001** |
| NRTIs + NNRTIs | 69 (46.9) | 36 (46.2) | 33 (47.8) |  |
| NRTIs + PIs | 12 (8.2) | 5 (6.4) | 7 (10.1) |  |
| NRTIs + INSTIs | 11 (7.5) | 3 (3.8) | 8 (11.6) |  |
| Untreated | 18 (12.2) | 10 (12.8) | 8 (11.6) |  |
| Inconclusive | 37 (25.2) | 24 (30.8) | 13 (18.8) |  |
| **Treatment received** |  |  |  | **＜0.001** |
| CHOP | 15 (10.2) | 2 (2.6) | 13 (18.8) |  |
| R-CHOP | 14 (9.5) | 1 (1.3) | 13 (18.8) |  |
| DA-EPOCH | 92 (62.6) | 65 (83.3) | 27 (39.1) |  |
| R-DA-EPOCH | 6 (4.1) | 3 (3.8) | 3 (4.3) |  |
| Other | 4 (2.7) | - | 4 (5.8) |  |
| Untreated | 16 (10.9) | 7 (9.0) | 9 (13.0) |  |
| **Treatment outcomes** |  |  |  | **＜0.001** |
| CR | 59 (40.1) | 24 (30.8) | 35 (50.7) |  |
| PR | 11 (7.5) | 3 (3.8) | 8 (11.6) |  |
| SD | 29 (19.7) | 18 (23.1) | 11 (15.9) |  |
| PD | 19 (12.9) | 12 (15.4) | 7 (10.1) |  |
| Untreated | 14 (9.5) | 7 (9.0) | 7 (10.1) |  |
| Death | 15 (10.2) | 14 (17.9) | 1 (1.4) |  |

GCB, germinal center B-cell-like; DE, MYC/BCL2 double-expression; EBER, Epstein-Barr virus-encoded small RNA; cART, combined antiretroviral therapy; NRTIs, nucleoside reverse transcriptase inhibitors; NNRTIs, non-nucleoside reverse transcriptase inhibitors; PIs, protease inhibitors; INSTIs, integrase strand transfer inhibitors; CHOP, cyclophosphamide, doxorubicin, vincristine, and prednisone; R, rituximab; DA-EPOCH, dose-adjusted etoposide, vincristine, cyclophosphamide, prednisone, and doxorubicin; CR, complete response; PR, partial response; SD, stable disease; PD, progressive disease.

**SUPPLEMENTARY FIGURE LEGENDS**

**
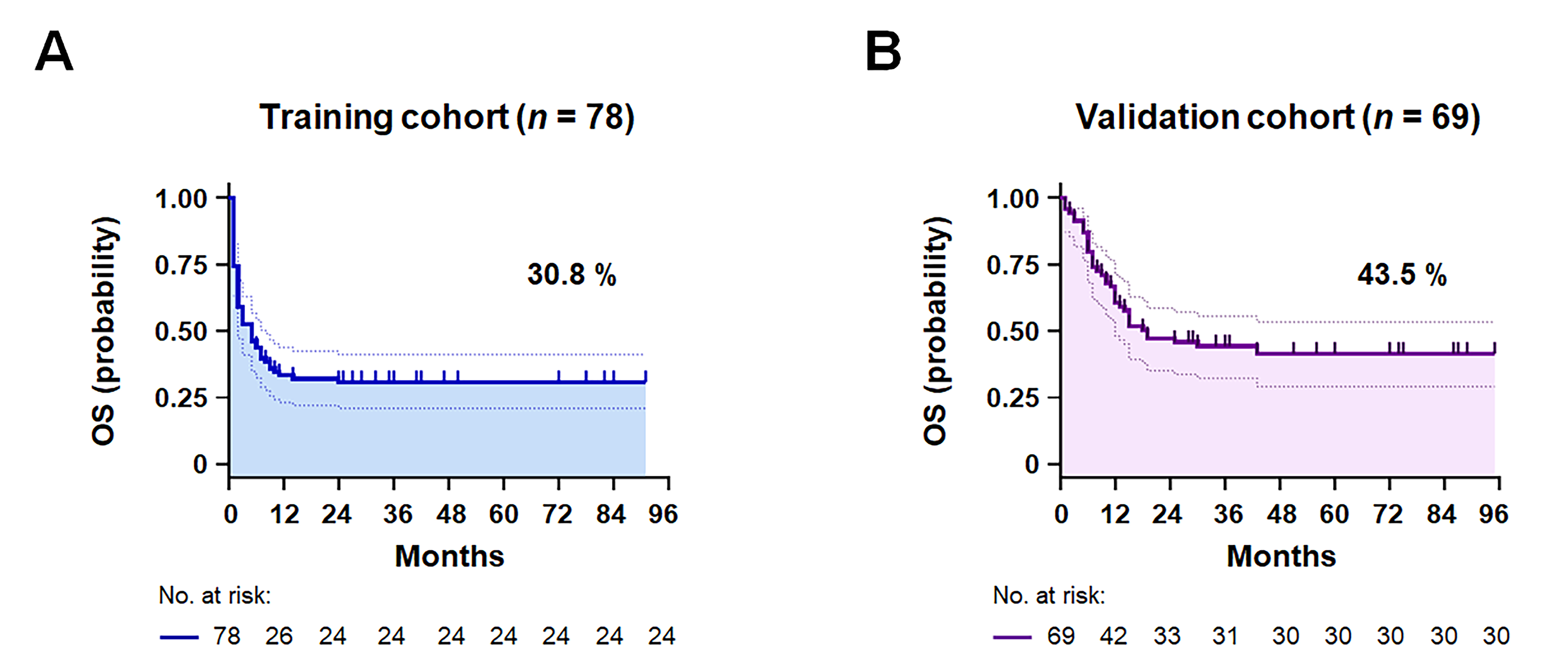
**

**SUPPLEMENTARY FIGURE S1** Survival analysis. Kaplan–Meier estimate of overall survival in the training (A) and the validation (B) cohorts.

**
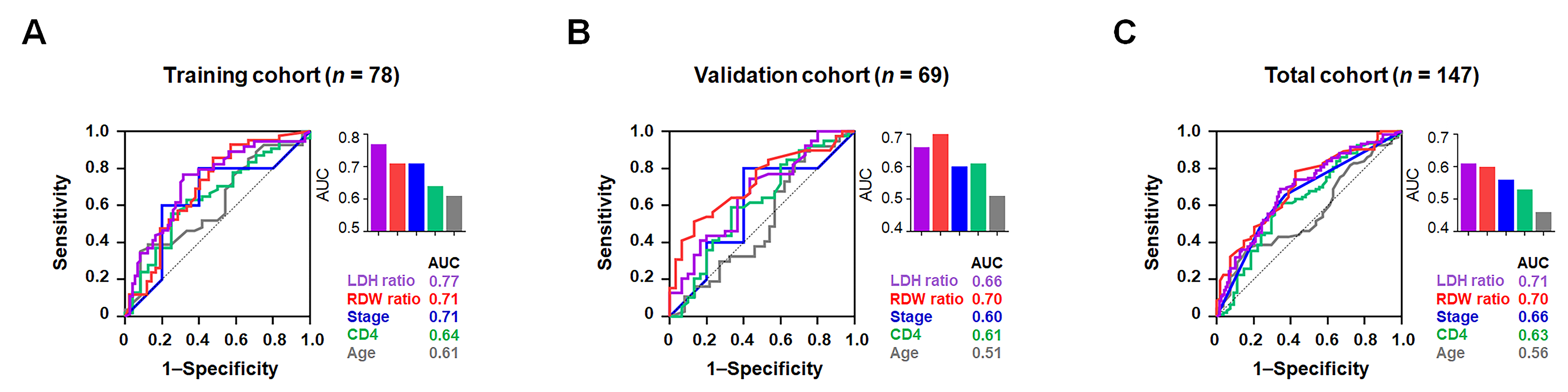
**

**SUPPLEMENTAL FIGURE S2** The receiver operating characteristic (ROC) curves and the area under curves (AUC) of LDH ratio, RDW ratio, Ann Arbor stage, CD4, and age in the training (A), the validation (B), and the total (C) cohorts.

**
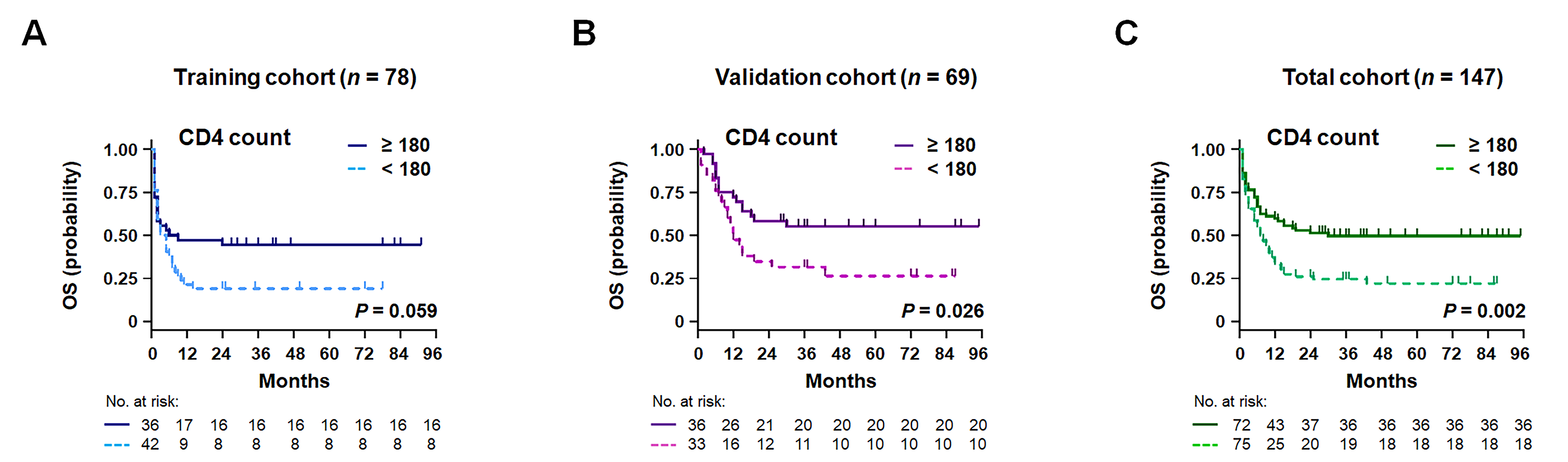
**

**SUPPLEMENTAL FIGURE S3** Survival curves stratified by the CD4 count. Kaplan–Meier analysis of overall survival for CD4 count in the training (A), the validation (B), and the total (C) cohorts.
